# Coordination and divergence in community assembly processes across co-occurring microbial groups separated by cell size

**DOI:** 10.3389/fmicb.2023.1166322

**Published:** 2023-06-02

**Authors:** Xinghao Li, James C. Stegen, Yuhe Yu, Jie Huang

**Affiliations:** ^1^Hubei Key Laboratory of Regional Development and Environmental Response, Hubei Engineering Research Center for Rural Drinking Water Safety, Hubei University, Wuhan, China; ^2^Donghu Experimental Station of Lake Ecosystems, Key Laboratory of Aquatic Biodiversity and Conservation of Chinese Academy of Sciences, Institute of Hydrobiology, Chinese Academy of Sciences, Wuhan, China; ^3^Fundamental and Computational Sciences Directorate, Biological Sciences Division, Pacific Northwest National Laboratory, Richland, WA, United States

**Keywords:** ecological process, cell size, high-throughput sequencing, prokaryotes, microbial eukaryotes

## Abstract

Setting the pace of life and constraining the role of members in food webs, body size can affect the structure and dynamics of communities across multiple scales of biological organization (e.g., from the individual to the ecosystem). However, its effects on shaping microbial communities, as well as underlying assembly processes, remain poorly known. Here, we analyzed microbial diversity in the largest urban lake in China and disentangled the ecological processes governing microbial eukaryotes and prokaryotes using 16S and 18S amplicon sequencing. We found that pico/nano-eukaryotes (0.22−20 μm) and micro-eukaryotes (20−200 μm) showed significant differences in terms of both community composition and assembly processes even though they were characterized by similar phylotype diversity. We also found scale dependencies whereby micro-eukaryotes were strongly governed by environmental selection at the local scale and dispersal limitation at the regional scale. Interestingly, it was the micro-eukaryotes, rather than the pico/nano-eukaryotes, that shared similar distribution and community assembly patterns with the prokaryotes. This indicated that assembly processes of eukaryotes may be coupled or decoupled from prokaryotes’ assembly processes based on eukaryote cell size. While the results support the important influence of cell size, there may be other factors leading to different levels of assembly process coupling across size classes. Additional studies are needed to quantitatively parse the influence of cell size versus other factors as drivers of coordinated and divergent community assembly processes across microbial groups. Regardless of the governing mechanisms, our results show that there are clear patterns in how assembly processes are coupled across sub-communities defined by cell size. These size-structured patterns could be used to help predict shifts in microbial food webs in response to future disturbance.

## Introduction

Understanding the processes that structure microbial communities in ecosystems is a fundamental goal in ecology (Gleason, 1927; Stegen et al., 2012). Analytical frameworks have been developed to identify the ecological process (Stegen et al., 2013) and distribution patterns of microbial communities (Chen et al., 2020). Two types of processes, namely deterministic and stochastic, can influence microbial assembly (Dini-Andreote et al., 2015). Deterministic processes are generally associated with effects of abiotic conditions (environmental filtering) and biotic interactions (Vellend, 2010; Zhou and Ning, 2017). Stochastic processes include probabilistic dispersal and random changes in species abundances that are not the consequence of environmentally determined fitness (Rosindell et al., 2011).

Microbial community assembly was originally studied from a deterministic perspective (Lozupone and Knight, 2007; Chase and Myers, 2011), where empirical evidence shows that a variety of environmental factors such as temperature, pH, salinity, and organic carbon influence community establishment at different scales (Bell et al., 2009; Gilbert et al., 2012; Wagner et al., 2017). Recent studies, however, provide increasing support for a significant role of stochastic process in microbial systems (Stegen et al., 2013). These studies have resulted in the need to quantify the relative influence of deterministic and stochastic processes on community assembly (Ofiteru et al., 2010). However, most studies that examine assembly processes look at the whole community. The variation in assembly processes within subsets of communities has been rarely reported. To the best of our knowledge, sub-community analyses have primarily been performed on the basis of abundant and rare taxa, which has greatly improved our understanding of the ecological process governing microbial communities (Mo et al., 2018; Xue et al., 2018). Relative abundances are emergent properties of ecological assembly, however, and there is a need to further understand how assembly processes differ across intrinsic organismal attributes such as cell size.

Cell or body size is one of the most fundamental attributes of an organism and Elton proposed that the body sizes of organisms may strongly influence the ecological processes governing community structure (Elton, 1927). Biodiversity (e.g., the number of species) and species interactions (e.g., predator-prey feeding links within food webs) (Elton, 1927) are key attributes of ecological communities that can be influenced by organismal body sizes (Blanchard et al., 2017). More broadly, body size is considered a “master trait” that sets the pace of life, constrains the roles of organisms within food webs, and influences the structure and dynamics of various ecological networks (Trebilco et al., 2013; Blanchard et al., 2017). Measuring the relationships between cell size and microbial community assembly processes can, therefore, provide new insight into the fundamental principles governing microbial communities.

Here, we investigate community assembly across size fractionated samples from Lake Donghu, one of the largest freshwater lake complexes (approximately 33.9 km^2^) in China (Yan et al., 2017). Recently, a series of ecological improvements (e.g., the “Great Lake Donghu Ecological Water Network”) have been implemented to prevent the deterioration of water quality. This lake complex has experienced large inputs of nutrients and other pollutants from urban sewage and intensive aquaculture for nearly 30 years (Gong and Xie, 2001). Given the large spatial extent and large range of environmental conditions in Lake Donghu, we expected to observe significant influences of both dispersal limitation and environmental selection over microbial communities. Given that microbes interact with each other across size classes and taxonomic groups, we also expected assembly processes to be coordinated across the classes/groups studied here. Specific aims of this study were (i) to reveal the diversity and spatial distribution patterns of differently sized microbes across environmental gradients in a typical freshwater lake, and (ii) to analyze whether co-occurring groups of microbes with distinct cell sizes are assembled by distinct or coordinated ecological processes.

## Materials and methods

Microorganisms can be classified into different size ranges by filtration of water samples through membranes with different pore sizes (Hasegawa et al., 2003; Ma et al., 2017). The use of membrane filter techniques and DNA sequencing methods has improved the ability to study microbial diversity (Filker et al., 2016), especially in identifying rare, novel and atypical taxa (Liu et al., 2015).

### Size-based separation of microorganisms from water

**FIGURE 1:**
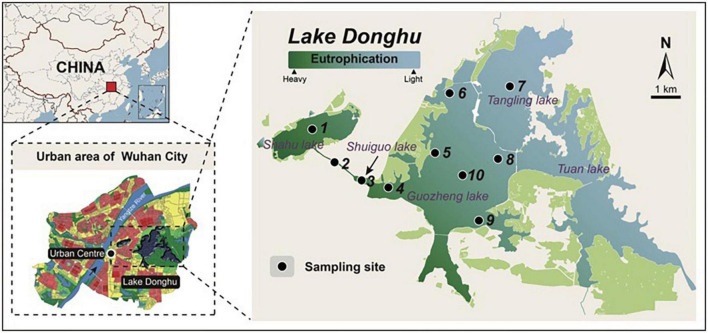
The location of sampling sites in Lake Donghu complex. Color bar indicates the environmental gradient (eutrophication) from the most heavily polluted region to the less polluted region.

Ten sites spanning an environmental gradient in the Lake Donghu complex were sampled in November 2016 according to the same protocol (Figure 1). Briefly, 5 L of water at three depths (i.e., surface, 1 m, 1.5 m or deeper depending on the depths of the sampling sites) were collected and filtered through a nylon net with 200 μm pore size. Filtrate from these three depths was then pooled to represent the whole water column (Filker et al., 2016). The pooled water samples were processed immediately in the laboratory, whereby 1 L of water was serially filtered through 20 and 0.22 μm pore size Durapore membranes (Millipore) using a vacuum pump. One 20 μm sized filter and 3−5 0.22 μm filters were obtained for each sample. Thus, two groups of microbes with different cell size ranges (i.e., micro-sized: 20∼200 μm and pico/nano-sized: 0.22∼20 μm, de Vargas et al., 2015) were obtained. For each site, three replicates were obtained and analyzed. After filtration, filters were immediately placed into cryovials and stored at −80°C until DNA extraction.

### Measurement of geographic distances and environmental variables

Geographic distance between each pair of sampling sites was estimated as the shortest distance over the Earth’s surface and was determined based on the longitude/latitude coordinates of each site (Supplementary Table 1) using the “distm” function in “geosphere” R package (v3.6.1) (R Core Team, 2019). Water temperature (Temp), dissolved oxygen concentration (DO), electrical conductivity (CON), and pH of the pooled water samples were measured using a multi-parameter probe (WTW 3430). Chemical parameters, including dissolved organic carbon (DOC), nitrate nitrogen (NO_3_-N), nitrite nitrogen (NO_2_-N), ammonium nitrogen (NH_4_-N), and orthophosphate (PO_4_-P) were determined as previously described (Simon et al., 2015). Chlorophyll a (Chl *a*) was also quantified by spectrophotometry after filtration of the pooled samples through a GF/C membrane (Whatman, NJ, USA).

### DNA extraction, PCR, and sequencing

Microbial genomic DNA was extracted using PowerWater DNA isolation kit (MoBio Laboratories, CA, USA) according to the manufacturer’s instructions. For detecting micro-eukaryotes, the hypervariable V4 region of the SSU rDNA was amplified using the primer pair EK-565F (5′-GCAGTTAAAAAGCTCGTAGT-3′) and primer 18 s-EUK-1134-R-UNonMet (5′-TTTAAGTTTCAGCCTTGCG-3′) (Bower et al., 2004; Simon et al., 2015). The amplification conditions were as follows: 35 cycles (95°C for 30 s, 55°C for 30 s and 72°C for 45 s), preceded by 3 min of denaturation at 95°C, and ending with a 10 min final extension step at 72°C. For detecting prokaryotes, the V4 region of the 16S rRNA gene was amplified with the primer set 515f (5′-GTGCCAGCMGCCGCGGTAA-3′) and 806r (5′-GGACTACHVGGGTWTCTAAT-3′) (Caporaso et al., 2011). Triplicate purified PCR products were pooled for library preparation. Sequencing was performed on the Illumina MiSeq PE300 platform (Illumina, San Diego, USA) at Majorbio Bio-Pharm Technology Co. Ltd., (Shanghai, China).

Raw reads were assembled using FLASH (Magoc and Salzberg, 2011) and quality-trimmed with Trimmomatic (Bolger et al., 2014). UCHIME was used to identify and remove chimeric sequences (Edgar et al., 2011). Quality-checked sequences were then clustered into operational taxonomic units (OTUs; 97% cutoff) using UPARSE v7.1 (Edgar, 2013).^1^ Taxonomic classification of the OTUs was conducted by Blast alignments (Altschul et al., 1990) against the Silva database version 138^2^ and PR2 database for microbial prokaryotes and eukaryotes, respectively. Fungal and metazoan OTUs were excluded prior to statistical analyses. Each sample was rarefied to the same sequence depth (*n* = 32,029 sequences for prokaryotes; *n* = 16,313 sequences for micro-sized eukaryotes; *n* = 13,975 sequences for pico/nano-sized eukaryotes).

### Description of the co-structure among subcommunities

Redundancy analysis (RDA) and canonical correspondence analysis (CCA) have been widely used to reveal variation in microbial community composition (Dray et al., 2003). These methods do not, however, directly couple the composition of co-occuring microbial groups (Palmer, 1993). To enable this coupling we used co-inertia analysis (COIA), which was specifically used to study relationships between each pair of subcommunity types (Dray et al., 2003).

### Analytical framework for studying community assembly processes

#### Testing for phylogenetic signals

To quantify the relative influences of different community assembly processes, we applied the analytical framework developed by Stegen et al. (2013) with slight modifications. This framework estimates the influences of variable selection, homogeneous selection, homogenizing dispersal, dispersal limitation and an “undominated” fraction. This framework uses phylogenetic and compositional turnover across communities. This requires an “OTU table” based on community composition and a “phylogenetic tree” based on OTU phylogenetic relationships.

**FIGURE 2:**
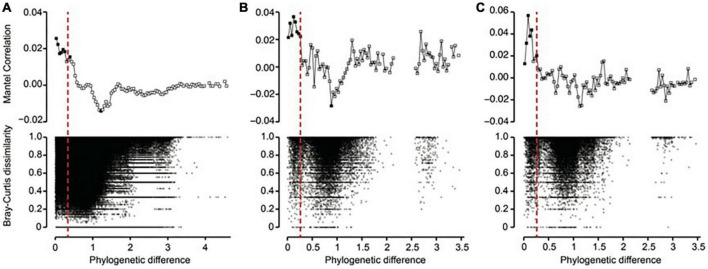
Phylogenetic Mantel correlogram showing significant phylogenetic signals across short phylogenetic distances. Solid and open symbols denote significant and non-significant correlations, respectively, relating the between-OTU version of Bray-Curtis distances of **(A)** prokaryotes, **(B)** pico/nano-eukaryotes (0.22–20 μm), and **(C)** micro-eukaryotes (20–200 μm) to between-OTU phylogenetic distances across a given phylogenetic distance. Significantly positive correlations indicate that Bray-Curtis distance between OTUs increases with their phylogenetic distance, but only across the phylogenetic distance class being evaluated (that is, there is phylogenetic signal in OTU distributions).

Using phylogenetic turnover to infer ecological processes requires the presence of phylogenetic signal, whereby more closely related taxa are more similar ecologically. To test for phylogenetic signal, we modified the procedures in Stegen et al. (2013) by using the between-OTU version of Bray-Curtis distances instead of the between-OTU niche differences to identify the microbial presence similarities. In this case, phylogenetic signal is indicated when the distribution patterns of closely related microbes are more similar to each other than to the distribution patterns of distant relatives. Nevertheless, it should be noted that lack of relationships using our method does not necessarily mean there is a lack of phylogenetic signal. This is because the method is more conservative than the standard approach described in Stegen et al. (2013). Therefore, if no significant relationships can be found using our method, further testing using niche values is recommended. To test for phylogenetic signal, a linear regression with permutation to control for non-independence of the data (i.e., Mantel test) was performed using the between-OTU version of Bray-Curtis distances and the phylogenetic distances. This showed that phylogenetic signal existed across relatively short phylogenetic distances, consistent with previous work (Figure 2). It is therefore most appropriate to quantify phylogenetic turnover among closest relatives (Stegen et al., 2012).

#### Quantifying community assembly processes

To characterize phylogenetic community composition at the local scale (for each individual community), we quantified the mean-nearest-taxon-distance (MNTD) and the nearest-taxon-index (NTI) using “mntd” and “ses.mntd” in the R package “picante.” NTI quantifies the number of standard deviations that the observed MNTD is from the mean of the null distribution (999 randomizations) (Stegen et al., 2012, 2013). Note that NTI is the negative of “mntd.obs.z” contained in the output of “ses.mntd” (Kembel et al., 2010). MNTD and NTI are calculated as follow.


$$\text{MNTD}=\sum_{i_{k}=1}^{n_{k}}{\text{f}}_{i_{k}}\,\text{min}\,\left({\text{Δ}}_{i_{k}\,j_{k}}\right)$$

where f_i_k__ is the relative abundance of OTU *i* in community k, *n*_*k*_ is the number of OTUs in *k*, and min (Δ_i_k_j_k__) is the minimum phylogenetic distance between OTU *i* and all other OTUs *j* that are also in *k*.


$$\text{NTI}=-1\,\left[\left({\mathrm{MNTD}}_{\text{obs}}-\bar{{\text{MNTD}}_{\text{null}}}\right)/\text{sd}\,\left({\text{MNTD}}_{\text{null}}\right)\right]$$

where MNTD_obs_ denotes the observed mean of phylogenetic distance and MNTD_null_ are null values of MNTD, and sd denotes the standard deviation. For a single community, NTI greater than +2 indicates coexisting taxa are more closely related than expected by chance (phylogenetic clustering). NTI less than −2 indicates coexisting taxa are more distantly related than expected by chance (phylogenetic overdispersion). A mean NTI taken across all communities that is significantly different from zero indicates clustering (NTI>0) or overdispersion (NTI<0) on average.

To further quantify phylogenetic turnover among closely related OTUs, but between community pairs, we used the beta-nearest taxon index (βNTI), which quantifies the degree that the beta-mean-nearest taxon distance (βMNTD) deviates from a null model expectation, as described in Stegen et al. (2013, 2015).


$$\beta \text{MNTD}=0.5\,\left[\sum_{i_{k}=1}^{n_{k}}{\text{f}}_{i_{k}}\,\text{min}\,\left({\text{Δ}}_{i_{k}\,j_{m}}\right)+\sum_{i_{m}=1}^{n_{m}}{\text{f}}_{i_{m}}\,\text{min}\,\left({\text{Δ}}_{i_{m}\,j_{k}}\right)\right]$$

where f_i_k__ is the relative abundance of OTU *i* in community *k*, *n*_*k*_ is the number of OTUs in *k* and min (Δ_i_m_j_k__) is the minimum phylogenetic distance between OTU *i* in community *k* and all OTUs *j* in community *m*. βMNTD was calculated using R function “comdistnt” (abundance. weighted = TRUE) (Kembel et al., 2010).


$$\beta \text{NTI}=\left(\beta {\text{MNTD}}_{\text{obs}}-\bar{\beta {\text{MNTD}}_{\text{null}}}\right)/\text{sd}\,\left(\beta {\text{MNTD}}_{\text{null}}\right)$$

where βMNTD_obs_ is observed βMNTD, βMNTD_null_ are null values of βMNTD, and sd indicates the standard deviation of the βMNTD_null_ distribution. We quantified βNTI for all pairwise comparisons, using a separate null model for each comparison.

Raup-Crick was used to identify whether the observed degree of turnover deviates from that expected for pairwise comparisons of phylogenetic community turnover that were not significant (see below) based on βNTI. These non-significant βNTI values indicate the potential dominance of stochastic processes (Chase et al., 2011; Stegen et al., 2015). Some applications of Raup-Crick do not consider OTU relative abundances, which carry information useful for understanding ecological processes. Here, we performed Raup-Crick as Stegen et al. (2015) described using Bray-Curtis dissimilarity to quantify compositional turnover. In short, a null distribution of Bray-Curtis values was first generated. Then, deviations between empirically observed Bray-Curtis and the null distribution were standardized to range from −1 to +1, which indicate whether local communities are more dissimilar (approaching 1), as dissimilar (approaching 0), or more similar (approaching −1), than expected by random chance. The resulting metric is referred as RC_*bray*_.

Overall, for a given community we estimated the relative influence of variable selection or homogeneous selection as the fraction of its comparisons to other communities with βNTI>+2 or βNTI<-2, respectively. Selection is excluded as the dominant process when | βNTI| <2; in these cases, RC_*bray*_>+0.95 or<-0.95 were taken as evidence that dispersal limitation or homogenizing dispersal, respectively, was the dominant process. For a given local community the relative influence of dispersal limitation was estimated as the fraction of its between-community comparisons with | βNTI| <2 and RC_*bray*_>+0.95. Similarly, the relative influence of homogenizing dispersal was estimated as the fraction of comparisons with | βNTI| <2 and RC_*bray*_<-0.95. The “undominated” scenario where | βNTI| <2 and | RC_*bray*_| <0.95 indicates that neither selection nor dispersal strongly drive compositional turnover and its relative contribution was estimated as the fraction of comparisons characterized by | βNTI| <2 and | RC_*bray*_ | <0.95.

## Results and discussion

To reveal variations in ecological processes between prokaryotes and microbial eukaryotes, as well as between eukaryotes with different cell sizes, we compared quantitative estimates of community assembly process across these three groups of microorganisms. Our results show that the assembly processes are clearly distinct across the three co-occurring microbial groups that are operationally defined by cell size. This is consistent with an influence of organismal cell size on community structure (Elton, 1927). Given the dominant influence of cell size over nearly all aspects of organismal ecology, cell size is likely to have direct or indirect causal influences over the assembly process patterns observed here.

### Phylotype diversity varied significantly across sub-communities

**FIGURE 3:**
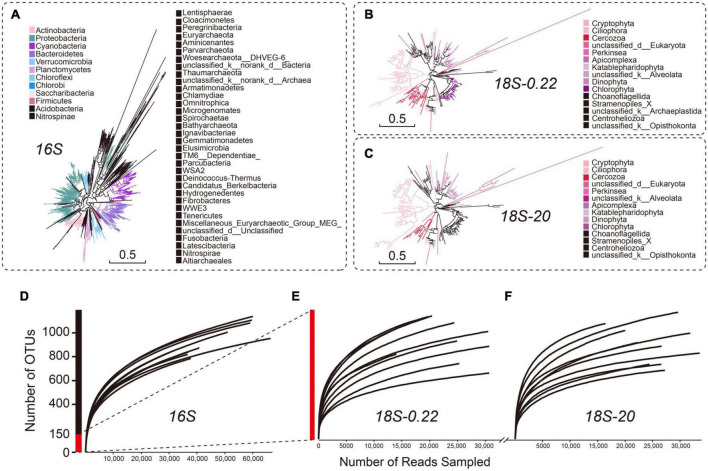
Broad view of the microbial diversity in different microbial groups. The phylogenetic trees of **(A)** prokaryotes, **(B)** pico/nano-eukaryotes (0.22–20 μm), and **(C)** micro-eukaryotes (20–200 μm) were constructed on OTU-level and were colored according to phyla. Only the top 10 abundant phyla are shown in color **(D–F)**. Rarefaction analyses predicted that all samples were sampled to near saturation. Prokaryotic diversity (estimated as number of OTUs) was much higher than eukaryotic diversity.

Using high-throughput sequencing analysis, we offer a comprehensive evaluation of microbial diversity across the sampled lake complex. We obtained between 67,978 and 119,131 high-quality reads for each sampling site after Illumina sequencing and data cleaning (Supplementary Table 2). We recovered more than 85% of the total phylotype diversity (measured as OTU richness based on full data sets) with an average of 1,036 ± 85 OTUs per sample (Supplementary Table 3). As expected, the prokaryotes (16 S) were generally more diverse (834 ± 71 OTUs) than the eukaryotes (101 ± 23 OTUs across all eukaryote samples/communities; One-way ANOVA, *P* < 0.05) (Figures 3A-C). Rarefaction analyses indicated that all samples were sampled to near saturation (Figures 3D-F). Consistent with previous studies (Yan et al., 2017; Xiao et al., 2019), our results suggest that Proteobacteria (33.38%; 685 out of 2052 OTUs), Bacteroidetes (14.57%; 299/2052 OTUs) and Cyanobacteria (10.48%; 215/2052 OTUs) were the three most diverse prokaryotic phyla. Other phyla accounting for more than 5% of the total phylotype diversity include Actinobacteria (9.06%) and Chloroflexi (5.12%).

Micro-eukaryotes (20−200 μm) and pico/nano-eukaryotes (0.22−20 μm) were characterized by similar phylotype diversity (i.e., 102 ± 27 OTUs and 99 ± 21 OTUs; One-way ANOVA, *P* > > 0.05), even though their community structures can be clearly distinguished from each other in the NMDS plot (phylogenetic composition, Supplementary Figure 1). Nearly 50% of the phylotype diversity showed unique patterns in both groups of micro-eukaryotes (Supplementary Figures 2A, B), which was also revealed by morphological observations (Supplementary Figure 3). Furthermore, the micro-eukaryotes were characterized by a high diversity of Ciliophora, Chlorophyta and Dinophyta, which accounted for up to 25.54%, 19.91%, and 9.52% of the total phylotype diversity (231 OTUs), respectively. The pico/nano-eukaryotes included Ciliophora, Chlorophyta, and Cercozoa, which accounted for up to 21.61%,16.53%, and 12.29% of the total phylotype diversity (236 OTUs), respectively.

### The sub-communities co-varied with each other, potentially due to trophic interactions

**FIGURE 4:**
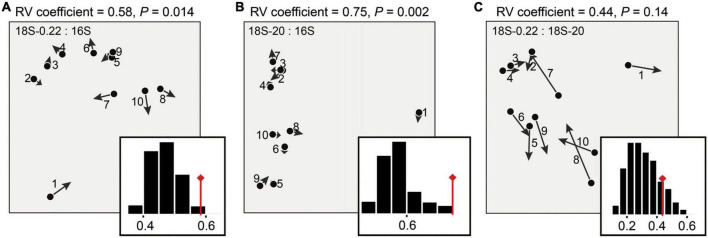
Description of the co-structure between the microbial groups. Co-inertia analysis (COIA) was applied to estimate the relationship **(A)** between prokaryotes and pico/nano-eukaryotes (0.22–20 μm), **(B)** between prokaryotes and micro-eukaryotes (20–200 μm), and **(C)** between pico/nano-eukaryotes and micro-eukarytoes. Each arrow of the COIA plots corresponds to a single sampling site, denoting the difference on the community structures between the compared microbial groups. Eigenvalues corresponding to the two co-inertia axes are shown in the histograms. Significance of co-structure was assessed by a Monte-Carlo test (RV coefficient, *P*).

Differences in eukaryotic community structures were visualized by NMDS ordination (Stress: 0.035), in which micro-eukaryotes and pico/nano-eukaryotes formed two distinct clusters (PERMANOVA, *P* < 0.05, for details see Supplementary Figure 1 and Supplementary Table 4). As expected, we also successfully captured the known co-variation between eukaryotic and prokaryotic communities (Figure 4; Monte-Carlo Test, *P* < 0.05). This is potentially explained by their close relationships within aquatic microbial food webs (Sherr and Sherr, 2002), whereby prokaryotes generally act as the main food resource of micro-eukaryotes and thus strongly shape the microbial eukaryotic distribution via bottom-up effects (Kuparinen and Bjornsen, 1992). More importantly, we found that the co-variation differed across cell size classes as the micro-eukaryotes exhibited a closer relationship with the prokaryotes (*R*^2^ = 0.75) than the pico/nano-eukaryotes did (*R*^2^ = 0.58) (Figures 4A, B). This result, along with studies on cladocera (Gliwicz, 1990; Rizo et al., 2019) and copepoda (Viitasalo et al., 1995), highlights the importance of cell size effects within aquatic microbial food webs. This pattern may be due to direct and indirect influences of cell size because cell size influences many aspects of organismal ecology (e.g., population abundance, growth rate, spatial niche) (Woodward et al., 2005). In terms of a direct influence of cell size, the most likely is via size-structured predator-prey feeding links (Trebilco et al., 2013). That is, close trophic interactions between microbial eukaryotes and prokaryotes likely only took place when the eukaryotes were big enough to be predators.

### The influence of deterministic assembly on local composition varied across sub-communities

**FIGURE 5:**
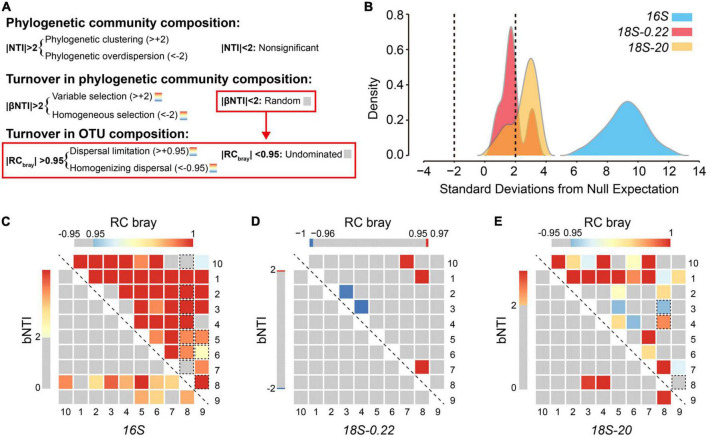
The relative influence of different ecological processes on microbial communities. **(A)** Summary of the analytical framework developed by Stegen et al. (2012, 2013). Local-scale phylogenetic community composition was characterized using the nearest-taxon-index (NTI). Turnover in phylogenetic community composition and OTU composition were quantified using the beta nearest-taxon-index (βNTI) and Bray-Curtis based Raup Crick (RC_bray_), respectively. **(B)** Kernel density estimates for distributions of abundance weighted NTI. As indicated by the dashed lines, individual values below –2 (negative) or above +2 (positive) are statistically significant, revealing phylogenetic overdispersion or clustering, respectively. Values of βNTI and RC_bray_ are shown using heatmaps for **(C)** prokaryotes, **(D)** pico/nano-eukaryotes (0.22–20 μm), and **(E)** micro-eukaryotes (20–200 μm). Values are indicated by color bars. Insignificant values are indicated as gray. Significant values are indicated in red or blue. For RC_bray_, rectangles surrounded by a dashed line indicate that the corresponding βNTI value was significant.

NTI across all local communities was significantly different from zero (*t*_30_ = 7.0, *P* < 0.0001), with a mean of +4.46 (Figure 5). This result, coupled with testing for phylogenetic signal, revealed that, in the Lake Donghu complex, closely related and ecologically similar taxa coexist to a greater degree than expected by chance (Figure 5A). Furthermore, the mean values of NTI varied significantly across different groups of microbes (One-way ANOVA, between the two eukaryotic groups: *F* = 4.6, *P* = 0.045; Overall: *F* = 162.5, *P* < 0.0001). One of the key inferences is that the relative influences of deterministic and stochastic processes over community structure at the local scale was different among the three microbial sub-communities.

Deterministic environmental filtering had a particularly strong influence over prokaryotic community composition, as indicated by an NTI distribution stretching from +4 to +14 with a median of +9.38 (Figure 5B). These NTI values are high in an absolute sense and are also high relative to the other two sub-communities. This provides good evidence that membership within the prokaryotic communities in Lake Donghu was strongly limited by abiotic and/or biotic factors. We suggest that top-down forces imposed by eukaryotes are one such biotic factor that likely played an important role. The microbial eukaryotic communities were also largely shaped by environmental filtering, but NTI values were smaller relative to the prokaryotes and varied across the two cell-sized classes. The micro-sized eukaryotes experienced stronger influences of environmental filtering with 70% of local communities having significant NTI values, while only 30% of pico/nano-sized eukaryotic communities had significant NTI values. These results combined with the co-variation analyses indicate a highly deterministic sub-system based on interactions between prokaryotes and micro-eukaryotes, and with pico/nano-eukaryotes assembling more stochastically and independently of this deterministic sub-system.

### Pico/nano-eukaryotes assembled independently from the other two sub-communities

To uncover the assembly processes influencing microbial community we coupled βNTI with RC_*bray*_ (Stegen et al., 2013). We examined 270 pairwise comparisons (135 for βNTI and 135 for RC_*bray*_) to quantify microbial community turnover in the Lake Donghu complex (Figures 5C-E). As previously shown, we found that environmental filtering has detectable influences on microbial assembly (Andersson et al., 2010; Ofiteru et al., 2010; Dini-Andreote et al., 2015). However, most of the βNTI values (122/135) were consistent with stochastic turnover (−2<βNTI<+2). We then applied RC_*bray*_ to pairwise comparisons with phylogenetic turnover that did not deviate from the null expectation (i.e., | βNTI| <2). About 50% (i.e., 63/122) of these pairwise comparisons did not deviate from the null expectation (i.e., | RC_*bray*_| <0.95). As such, the magnitude of community turnover in 47% of all pairwise comparisons (63/135) was not dominated by stochasticity or determinism.

These turnover results contrast with the within-community analyses that indicated strong influences of deterministic assembly. For example, only 7% (i.e., 3/45) of phylogenetic community turnover in the micro-sized eukaryotes was primarily due to environmental filtering (| βNTI| >2). This fraction, however, is much smaller than the fraction of significant NTI values (70%). Similar trends were also observed for the prokaryotes whereby 22% of turnover was deterministic vs. 100% determinism at the within-community scale. This suggests that deterministic processes strongly influenced local community composition, while stochastic processes have a stronger influence on community turnover (Mo et al., 2018).

While nearly half of all pairwise comparisons were not dominated by a single type of process, we found that dispersal limitation was the primary driver for 51% of community turnover in micro-sized eukaryotes, and the “undominated” fraction had an important but secondary role (42%) (Figure 5E). Similar results were observed for prokaryotes (Figure 5C), which were also strongly governed by dispersal limitation (∼69%). These results, combined with the significant relationships between community dissimilarity and geographic distance (Supplementary Figure 4), suggest that larger spatial distance increase the influence of dispersal limitation in the sampled lake complex.

These findings contrast with the classic paradigm that “all microbes are everywhere.” Moreover, the dispersal limitation effect on structuring microbial communities may also be related to the features of the studied lake system, which has been divided into several lake regions by artificial dykes. Interestingly, we noted that over 90% of spatial turnover in the community composition of the pico/nano-eukaryotes was undominated. That is, neither deterministic selection or spatial processes had a strong or consistent influence in the assembly of pico/nano-eukaryotes. This can be due to many reasons such as weak selection, or selective processes that change frequently through space or time, and/or spatially variable rates of dispersal. The much larger undominated fraction for the pico/nano-eukaryotes, relative to the other two sub-communities, provides further evidence that community assembly of the pico/nano-eukaryotes operated independently from the other two sub-communities.

## Conclusion

This study showed that co-occurring groups of microbes that are operationally defined by cell size are governed by divergent assembly processes. However, we also observed multiple patterns indicating that the community assembly processes influencing micro-eukaryotes are linked to community assembly processes influencing prokaryotes. We propose that this link is due, in part, to size-structured trophic interactions that lead to within-community deterministic assembly. We also provide multiple patterns indicating that the community assembly of pico/nano-eukaryotes is largely independent of the sub-system generated by the coupling between micro-eukaryotes and prokaryotes. This points to a potential influence of cell size whereby larger cell size enables trophic interactions with prokaryotes, and these interactions link community assembly of micro-eukaryotes and prokaryotes. While the results are consistent with an influence of cell size, there are likely other factors at work that may be indirectly associated with cell size or linked to other size-independent attributes of organismal ecology. Thus, additional studies should be conducted to quantitatively parse the direct and indirect influences of cell size over assembly processes, as well as the influences of size-independent mechanisms. Moreover, the results in this work add to a growing literature showing an important influence of dispersal limitation, which is now broadly recognized as working alongside environmental filtering to shape microbial communities.

## Data availability statement

Quality-checked assembled sequence data are deposited in NCBI Sequence Reads Archive under Bio-Project PRJNA848972.

## Author contributions

JH and YY conceived and designed the study. XL analyzed the data and wrote the first version of the manuscript. JS and JH wrote and revised the manuscript. All authors contributed to the article and approved the submitted version.

## Footnotes

1
http://drive5.com/uparse/

2
http://www.arb-silva.de
