# Supplementary material for: Coordination and divergence in community assembly processes across co-occurring microbial groups separated by cell size
Source: Front Microbiol. 2023 Jun 2;14:1166322. doi: 10.3389/fmicb.2023.1166322 (PMC10272581; doi:10.3389/fmicb.2023.1166322)
Supplement: Supplementary file 5 [file Data_Sheet_1.docx]

***Supplementary Information***

**Coordination and divergence in community assembly processes across co-occurring microbial groups separated by cell size**

Xinghao Li^1,2^, James C. Stegen^3^, Yuhe Yu^2^, Jie Huang^2^

^1^Hubei Key Laboratory of Regional Development and Environmental Response, Hubei Engineering Research Center for Rural Drinking Water Safety, Hubei University, Wuhan, China

^2^Donghu Experimental Station of Lake Ecosystems, Key Laboratory of Aquatic Biodiversity and Conservation of Chinese Academy of Sciences, Institute of Hydrobiology, Chinese Academy of Sciences, Wuhan, China

^3^Fundamental and Computational Sciences Directorate, Biological Sciences Division, Pacific Northwest National Laboratory, Richland, WA, United States

**Corresponding author:**

Jie Huang, jhuang@ihb.ac.cn

This supplementary information includes:

Fig. S1

Fig. S2

Fig. S3

Fig. S4

**Note that supplementary tables are respectively provided in four files in ‘.xls’ format.**


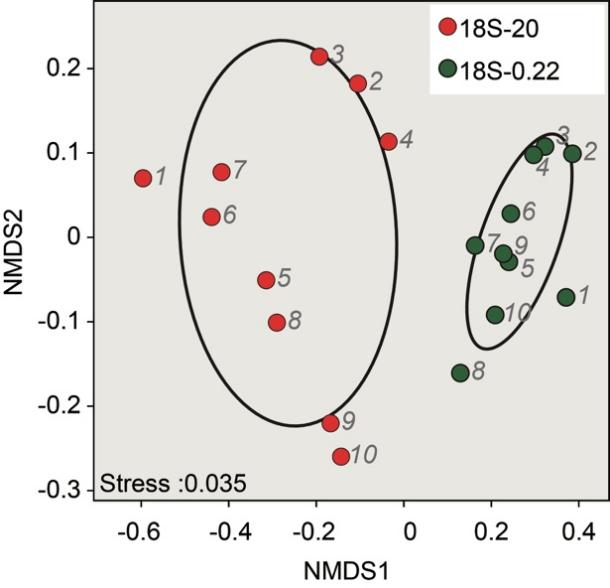


Fig. S1 Non-metric multidimensional scaling (NMDS) ordination of Bray-Curtis distance shows the dissimilarity of the micro-eukaryotic communities.


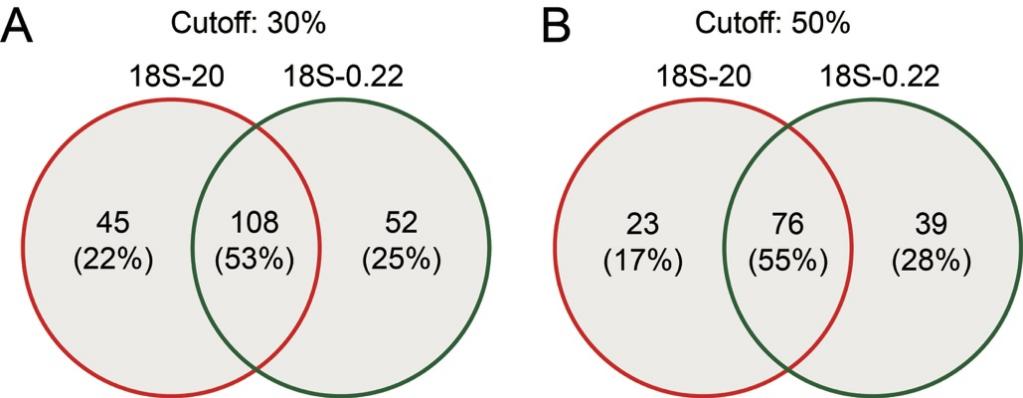


Fig. S2 Community dissimilarity of micro-eukaryotes in two different body size ranges. Venn diagram showing the number of shared and unique OTUs among the samples of differently sized micro-eukaryotes at (**A**) 30% and (**B**) 50% cutoff level. OTUs present only in at least 30% or 50% of samples in one group were considered unique to that group.


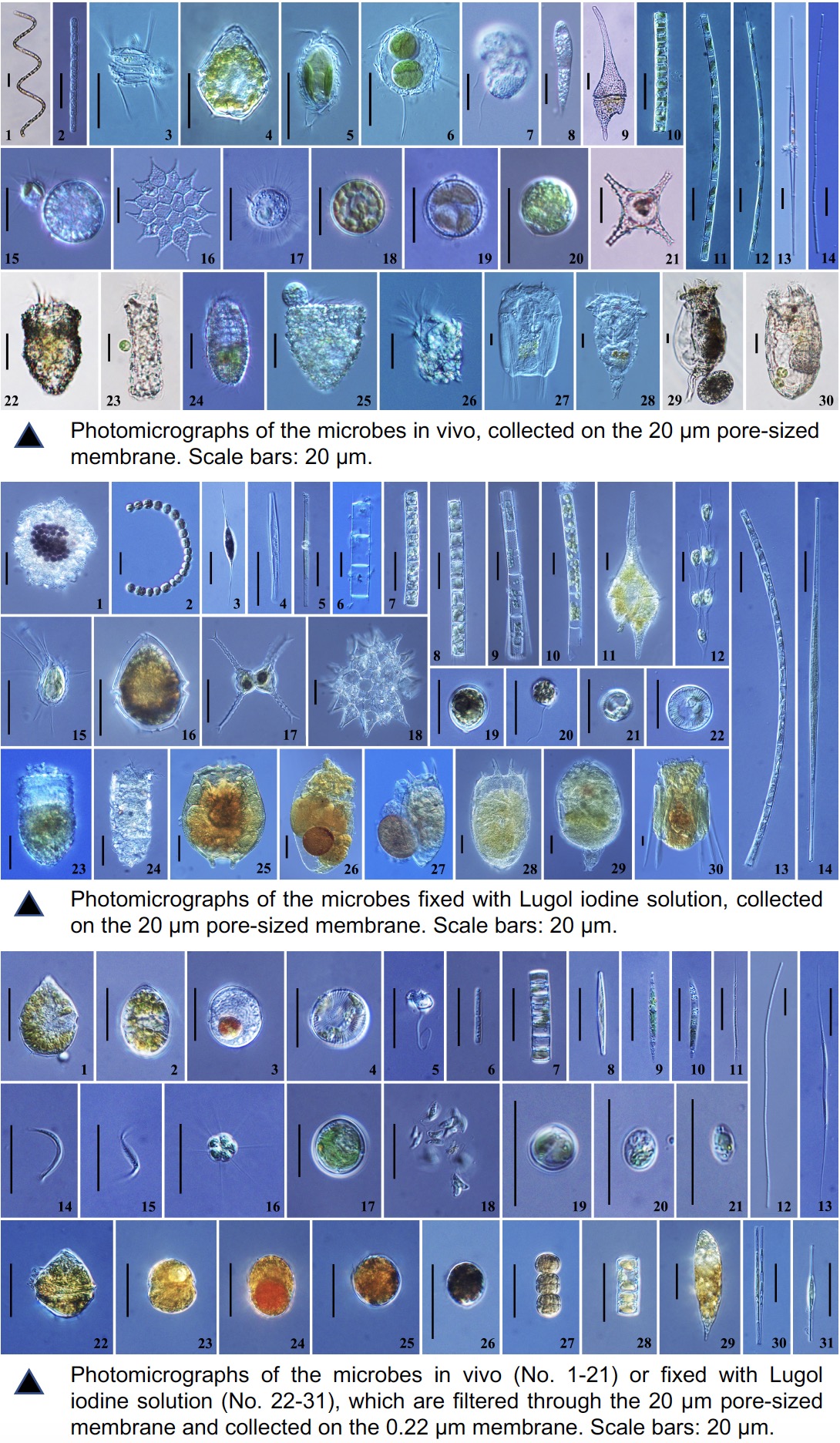
Fig. S3 Overview of the microbes that were collected using different pore-sized membrane and observed via microscopy.


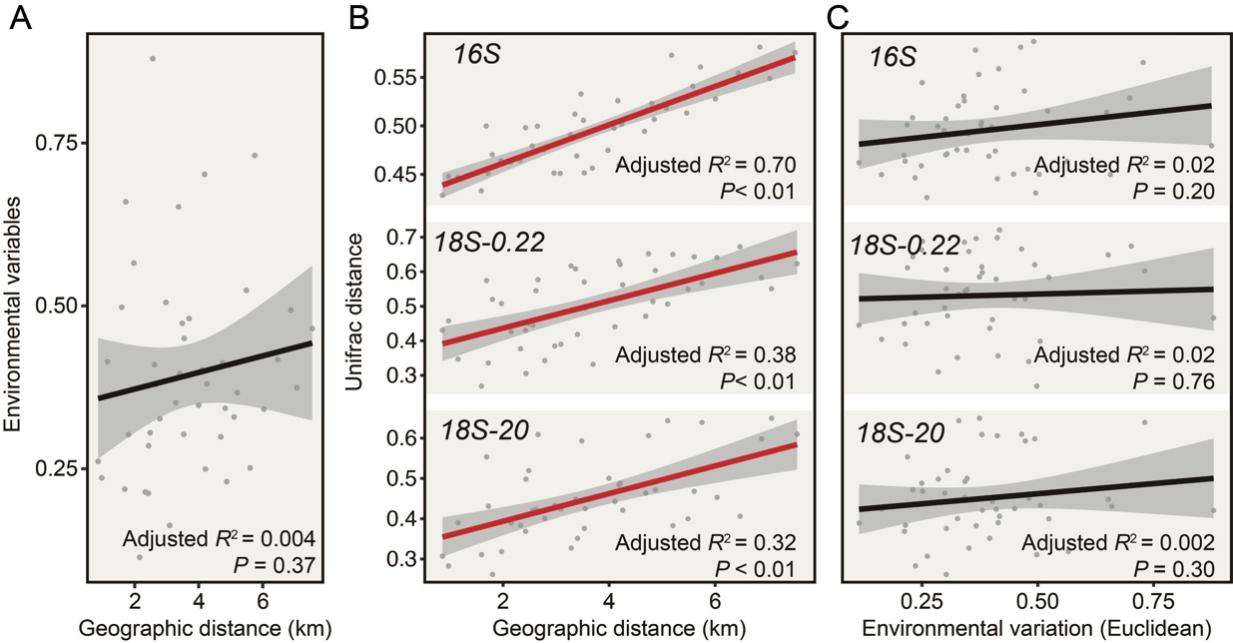


Fig. S4 Relationships between geographic distance, environmental factors, and microbial community. (**A**) Spearman’s rank correlation between the log-transformed geographic distance and the Euclidean distance of environmental variables. (**B** and **C**) Unifrac distance of microbial subcommunities are respectively correlated with geographic distance and the Euclidean distance of environmental variables. Lines in red represent significant correlations (*P*<0.05).
